# Endogenous programmed death ligand-1 restrains the development and onset of Sjӧgren’s syndrome in non-obese diabetic mice

**DOI:** 10.1038/srep39105

**Published:** 2016-12-14

**Authors:** Jing Zhou, Jun-O. Jin, Toshihisa Kawai, Qing Yu

**Affiliations:** 1 Department of Immunology and Infectious Diseases, the Forsyth Institute, 245 First Street, Cambridge, MA 02142, USA; 2 Department of Oral Medicine, Infection and Immunity, Harvard School of Dental Medicine, 188 Longwood Avenue, Boston, MA 02115, USA

## Abstract

Programmed death-ligand 1 (PD-L1) down-modulates various immune responses by engaging the co-inhibitory receptor programmed death-1. Expression of PD-L1 and programmed death-1 is elevated in the salivary glands of patients with Sjögren’s syndrome (SS). The objective of this study is to define the role of endogenous PD-L1 in SS pathogenesis in non-obese diabetic (NOD) mouse model of this disease. We inhibited endogenous PD-L1 function by intraperitoneal administration of a blocking antibody to 6 week-old female NOD/ShiLtJ mice repeatedly during a 9-day period. PD-L1 blockade accelerated leukocyte infiltration and caspase-3 activation in the submandibular gland (SMG), production of antinuclear and anti-M3 muscarinic acetylcholine receptor (M3R) autoantibodies and impairment of saliva secretion, indicative of accelerated development and onset of SS. The effect of PD-L1 blockade was associated with increased T- and B cells and T helper 1 cytokine IFN-γ in the SMG. Local administration of exogenous IFN-γ to the SMG led to impaired salivary secretion accompanied by down-regulation of aquaporin 5 and an increase in anti-M3R autoantibodies. Conversely, neutralization of IFN-γ markedly improved salivary secretion and aquaporin 5 expression in anti-PD-L1-treated NOD/ShiLtJ mice. Hence, endogenous PD-L1 hinders the development and onset of SS in NOD mice, in part by suppressing IFN-γ production.

Sjӧgren’s syndrome (SS) is a systemic autoimmune disease affecting an estimated 2–4 million Americans^1^. It is characterized by lymphocytic infiltration of exocrine glands, particularly salivary and lacrimal glands, production of autoantibodies, exocrine gland destruction and secretory dysfunction^2^,^3^,^4^. The hallmark symptoms of SS are dry mouth and dry eyes^2^,^4^. It also frequently affects many other organs and causes an array of symptoms and health complications, including B cell lymphoma^2^,^4^,^5^. SS can occur alone as primary SS or in conjunction with other inflammatory connective tissue diseases as secondary SS^6^. T and B cells are the main immune cell populations that infiltrate exocrine glands and are essentially required for the development and onset of SS^7^,^8^,^9^,^10^. T cell-derived cytokines, including IFN-γ, IL-4, and IL-17, signature cytokines for the major T helper (Th) cell subsets, play indispensable roles in the pathogenesis of SS by promoting tissue inflammation and destruction and facilitating B cell activation and autoantibody production^1^,^11^. SS patients exhibit elevated Th1 cytokine IFN-γ levels and enhanced Th1 response in salivary glands and saliva compared to non-SS sicca patients^12^,^13^,^14^,^15^. Moreover, the degree of local Th1 response strongly correlates with the pathologies of the salivary glands^16^. Importantly, studies with IFN-γ-deficient mice demonstrate an indispensable role of this cytokine in the development and onset of SS^17^. IFN-γ contributes to the pathogenesis of SS by multiple mechanisms. It can induce tissue apoptosis, especially in cooperation with TNF-α^18^,^19^,^20^. It induces expression of chemoattractants CXCL9 and -10 in salivary gland tissues, thereby promoting the tissue recruitment of CXCR3-expressing T cells, which are predominantly Th1 and T cytotoxic (Tc) 1 cells^21^. IFN-γ also enhances the antigen presenting function of the salivary gland cells to facilitate immune activation^7^,^22^. Therefore, endogenous immunoregulatory pathways or exogenous immune-suppressive approaches that can attenuate Th1/Tc1 responses and IFN-γ production may have preventive or therapeutic potentials for SS disease.

A multitude of soluble factors and cell surface molecules are up-regulated during SS development, including both positive and negative regulators of the autoimmune responses and pathologies. The costimulatory pathway formed by programmed death-ligand 1 (PD-L1) and its receptor PD-1 plays a critical role in maintaining immune tolerance and limiting immune activation and tissue damage, predominantly by suppressing the differentiation, activation and IFN-γ production of Th1 and Tc1 cells^23^,^24^,^25^,^26^,^27^,^28^, as well as enhancing the differentiation and function of regulatory T (Treg) cells^29^. PD-1 is expressed on the surface of activated lymphocytes and antigen presenting cells (APCs)^30^. PD-L1 is constitutively expressed on resting lymphocytes and APCs and its expression is upregulated upon activation of these cells by various stimuli, including IFN-γ and TNF-α^31^,^32^,^33^,^34^. Apart from immune cells, PD-L1 is induced in various types of non-hematopoietic cells by pro-inflammatory cytokines including IFN-γ^35^,^36^, allowing it to exert immunoregulatory function in target organs of various autoimmune and inflammatory diseases. Hence, PD-L1-PD-1 pathway is activated as a result of immune activation and serves as a negative feedback mechanism that down-modulates T cell immune responses. Indeed, PD-L1 expression is elevated in the inflamed tissues of a number of autoimmune diseases, including type 1 diabetes, autoimmune encephalomyelitis (EAE), Crohn’s syndrome, and rheumatoid arthritis^29^,^37^,^38^, and the tissue-infiltrating T cells in these autoimmune disorders express surface PD-1^38^,^39^,^40^,^41^. Loss-of-function studies in mouse models demonstrate that the endogenous PD-L1-PD1 activities restrain the development and reduce the severity of lupus-like glomerulonephritis and arthritis, EAE, autoimmune diabetes and collagen-induced arthritis^29^,^42^,^43^. Moreover, enforced activation of PD-L1-PD-1 pathway impedes the development and reduces the severity of these diseases^44^,^45^. The disease-inhibiting effect of PD-L1-PD-1 is predominantly associated with reduced Th1 and Tc1 responses and impaired IFN-γ production^25^,^46^,^47^, and in some cases, a dampened Th17 responses^48^,^49^.

PD-L1 and PD-1 expression are elevated in salivary gland epithelial cells and salivary gland-infiltrating lymphocytes, respectively, in SS patients^35^, suggesting a potential immune-suppressive and disease-inhibiting role of PD-L1-PD-1 pathway in this disease. In the present study, we investigated the role of endogenous PD-L1 in SS by inhibiting its function in non-obese diabetic (NOD) mice, a widely used model of SS, and demonstrated an inhibitory effect of endogenous PD-L1 that hinders the development and onset of this disease.

## Results

### Expression of PD-L1 and PD-1 in the submandibular glands (SMG) of NOD/ShiLtJ mice increases during the development of SS-like disease

**Figure 1:**
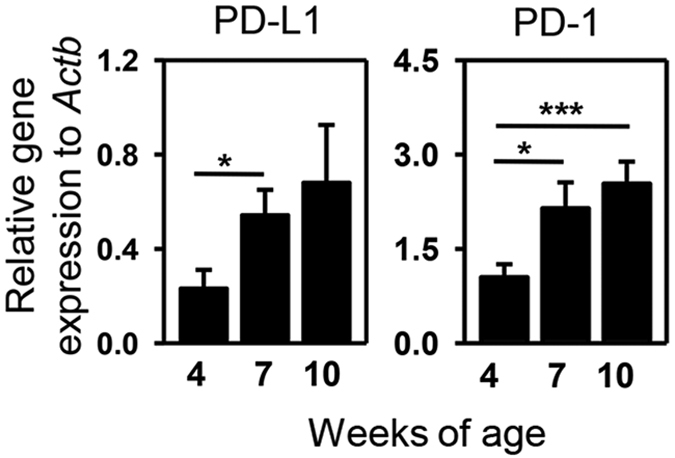
Expression level of PD-L1 and its receptor PD-1 in the SMG of NOD/ShiLtJ mice. Real-time PCR analysis of PD-L1 and PD-1 mRNA levels in the SMG of NOD/ShiLtJ mice aged 4, 7 and 10 weeks, presented relative to that of β-actin. Data are the average of the analyses of 4–7 mice for each group. Error bars represent the standard error of mean (SEM). *P < 0.05, **P < 0.01; ***P < 0.001.

Increased PD-L1 and PD-1 expressions are shown in patients with SS^35^. To examine whether PD-L1 and PD-1 expression is elevated during the development of SS-like disease in NOD/ShiLtJ mice, we determined their mRNA levels in the SMG from these mice at various ages. We first determined the time course of SS development in the female NOD/ShiLtJ mice by examining mice aged 4-, 7-, 10- and 12 weeks. The results showed that the disease onset started around 10 weeks of age in the great majority of these mice, based on the presence of leukocyte foci in the SMG and antinuclear antibodies (ANA) in the serum, as well as an impaired stimulated salivary flow rate compared to mice aged 4 weeks and control Balb/c mice (data not shown). We thus assessed PD-L1 and PD-1 gene expression at 4-, 7- and 10 weeks of age by real-time PCR analysis, which showed that the amounts of both PD-L1 and PD-1 mRNA in the SMG were significantly increased between age 4 and 7 weeks, and PD-1 mRNA levels were further increased between age 7 and 10 weeks (Fig. 1). Hence, PD-L1 and PD-1 expression in the SMG increases during the early phase of SS development in NOD/ShiLtJ mice, suggesting that PD-L1-PD-1 pathway may act as a negative feedback mechanism to suppress autoimmune responses and hinder the further development of this disease.

### Blockade of endogenous PD-L1 accelerates the development of characteristic SS-like pathologies in NOD/ShiLtJ mice

**Figure 2:**
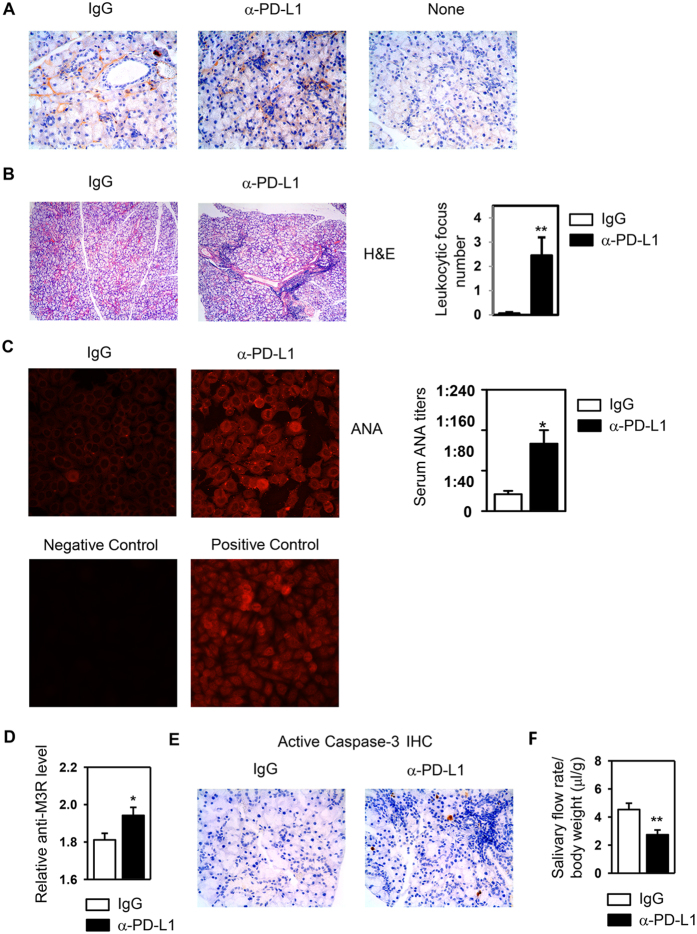
Blockade of PD-L1 accelerates the appearance of SS-like pathologies. NOD/ShiLtJ mice received injections of rat anti-mouse PD-L1 antibody and control rat IgG2b every other day for a total of 4 times. (**A**) The presence of the injected antibody and control IgG in the SMG was examined by horseradish peroxidase (HRP) based immunohistochemical staining with an anti-rat IgG antibody. SMG sections from mice not receiving any injections were used as negative controls. Original magnification: ×400. Data are representative of 6–7 mice for each group. (**B**) H&E staining of SMG sections with original magnification of ×200 (left), and mean leukocyte focus number in the SMG (right). (**C**) Detection of serum ANA (original magnification: ×400) and determination of its titers (right panel). (**D**) Serum anti-M3R autoantibody level was determined by ELISA. (**E**) Immunohistochemical staining of cleaved, active caspase-3 in SMG sections. Original magnification: ×400. (**F**) Stimulated saliva flow rate normalized to body weight. Data are representative or the average of the analyses of 18–20 mice for each group. Error bars represent the SEM. *P < 0.05, **P < 0.01; ***P < 0.001.

To investigate whether endogenous PD-L1 expressed in the SMG affects the pathogenesis of SS, we intraperitoneally (*i.p.*)-administered 200 μg of an anti-PD-L1 antibody, which blocks the interaction between PD-L1 and its receptors, or the isotype control, rat IgG2b, into 6 week-old female NOD/ShiLtJ mice every other day for a total of 4 times and analyzed the disease profiles in these mice. To determine whether systemically injected anti-PD-L1 antibody was delivered into the target organ SMG, the presence of rat IgG2b in this organ was assessed by immunohistochemical staining. The positive staining of rat IgG2b was detected in the SMG of both anti-PD-L1- and rat IgG2b-treated mice, whereas no staining was observed in that of untreated control mice (Fig. 2A), indicating that the injected antibody was present in the SMG and could inhibit the function of PD-L1 expressed locally as intended. We next analyzed a series of hallmark pathologies of SS to determine the role of endogenous PD-L1 in the development of this disease. Histological analysis by H&E staining demonstrated that at the time of the analysis, only 5.5% of IgG-treated NOD/ShiLtJ mice had leukocytic foci in the SMG tissues, whereas 55% of anti-PD-L1-treated mice had foci, with the average focus number higher than 2 (Fig. 2B, Supplementary Figure S1 and Table S1). To assess whether PD-L1 blockade alters the production of ANA, we determined the presence of ANA in the serum by indirect immunofluorescence staining for mouse IgG that recognizes human epithelial (HEp-2) cell substrates. The results showed that 47% of IgG-treated mice were positive for serum ANA, and this percentage was increased to 81% in anti-PD-L1-treated mice. Moreover, among the ANA-positive mice, PD-L1 blockade led to an increase in the levels of ANA as indicated by higher fluorescence intensity of the staining (Fig. 2C). Autoantibodies against M3 muscarinic acetylcholine receptor (M3R) have been shown to interfere with normal salivary secretion in response to neurotransmitters^50^,^51^, and ELISA assay demonstrated that PD-L1 blockade led to increased serum anti-M3R levels in the NOD/ShiLtJ mice (Fig. 2D). Immunohistochemical staining of SMG sections showed that at the time of the analysis, IgG-treated NOD/ShiLtJ mice had very low levels of active caspase-3 in the SMG tissues (Fig. 2E). In contrast, most of the anti-PD-L1-injected mice exhibited caspase-3 activation in the SMG (Fig. 2E), suggestive of tissue apoptosis and damage that can contribute to the development of SS pathologies and the secretory dysfunction of the SMG. Finally, anti-PD-L1-treatment significantly decreased the stimulated saliva flow rate, indicating an impaired secretory function and clinical onset of SS (Fig. 2F). Hence, PD-L1 blockade accelerates the development and clinical onset of SS.

### PD-L1 blockade increases the number of T and B cells in the SMG

**Figure 3:**
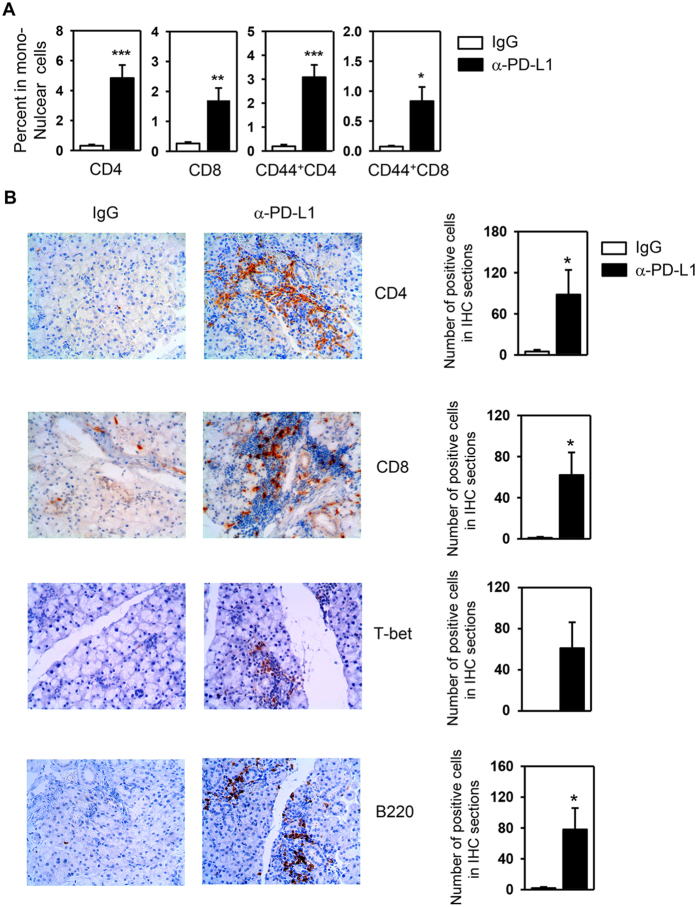
PD-L1 blockade increases the number of T and B cells and in the SMG. Anti-PD-L1 or control rat IgG was injected into 6 week-old NOD/ShiLtJ mice, every other day for a total of 4 times. (**A**) Flow cytometry of lymphocyte populations in SMG-infiltrating mononuclear cells. (**B**) Immunohistochemical staining of CD4, CD8, T-bet and B220 in SMG sections and the quantification of the number of positively-stained cells (right panels). Original magnification: ×400. Data are representative or the average of the analyses of 6–7 mice for each group. *P < 0.05, **P < 0.01; ***P < 0.001.

We subsequently examined the effect of PD-L1 blockade on lymphocyte numbers in the SMG. Flow cytometric analysis showed that the percentages of CD4^+^ and CD8^+^ T cells among the SMG-infiltrating mononuclear cells were markedly higher in anti-PD-L1-treated NOD/ShiLtJ mice than IgG2b-treated controls (Fig. 3A and Supplementary Figure S2). Moreover, the percentages of CD44^+^CD4 and CD44^+^CD8 T cells, which represented the effector or memory T cells, were also significantly increased by anti-PD-L1 treatment (Fig. 3A). Immunohistochemical staining of the SMG sections showed that a significant amount of CD4^+^, CD8^+^ and B220^+^ cells were present in the SMG of anti-PD-L1-treated mice, especially in the leukocytic foci (Fig. 3B). In contrast, very few of these cells were detected in the SMG from IgG-treated control mice, consistent with a lack of foci (Fig. 3B). In addition, the immunohistochemical staining provided evidence that anti-PD-L1 treatment increased the number of Th1 and Tc1 cells in the SMG, as indicated by increased amount of cells expressing T-bet, the signature transcription factor for Th1 and Tc1 subsets (Fig. 3B). In contrast, virtually no cells in the SMG expressed GATA3, the master regulator of Th2 cells (Supplementary Figure S3). Interestingly, the amount of cells that expressed Foxp3, the master regulator of Tregs, were significantly increased upon PD-L1 blockade, indicating that the promoting effect of anti-PD-L1 on SS development did not result from a decrease in Tregs (Supplementary Figure S3). In summary, the disease-accelerating effect of PD-L1 blockade on SS development is accompanied by increased T and B cells and elevated T-bet levels in the SMG.

### PD-L1 blockade increases the expression of Th1 signature molecules and lymphocyte chemoattractants in the SMG

**Figure 4:**
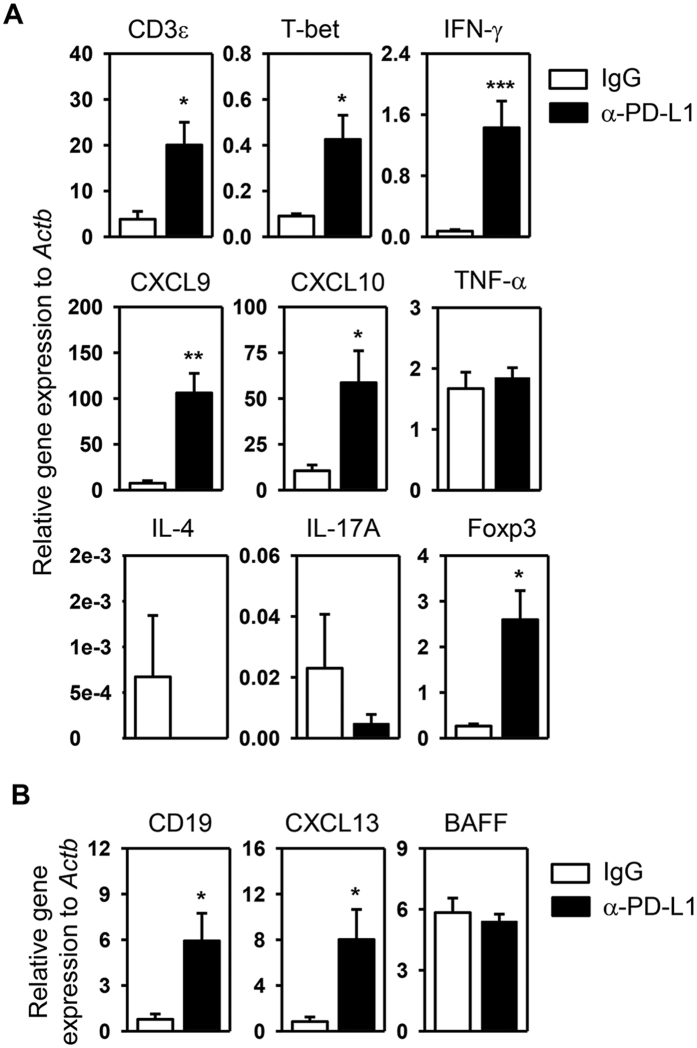
PD-L1 blockade increases expression of Th1 signature molecules and lymphocyte chemoattractants in the SMG. Anti-PD-L1 or control rat IgG was injected into NOD/ShiLtJ mice as described earlier. Real-time PCR analysis of (**A**) T cell-associated gene expression and (**B**) B cell-associated gene expression in the SMG, presented relative to that of β-actin. Data are the average of the analyses of 6–7 mice for each group. *P < 0.05, **P < 0.01; ***P < 0.001.

To further understand the effect of PD-L1 blockade on the autoimmune responses in the SMG, we assessed the expression of hallmark cytokines for various effector T cell subsets. In accordance with increased T cell numbers and T-bet levels in the SMG, mRNA levels of CD3ε, IFN-γ and T-bet were all elevated upon PD-L1 blockade (Fig. 4A). In contrast, PD-L1 blockade did not increase the amount of IL-4 and IL-17A mRNA, suggesting that endogenous PD-L1 plays a protective role in SS by suppressing Th1 and Tc1 type responses but not Th2 or Th17 responses (Fig. 4A). Consistent with the increase in Foxp3-expressing cells in the SMG as determined by immunohistochemical staining, the transcription level of Foxp3 was also increased by PD-L1 blockade (Fig. 4A). We next assessed if PD-L1 blockade affects the expression of CXCR3 ligands CXCL9 and -10, which are chemokines that can facilitate the recruitment of CXCR3-expressing Th1 and Tc1 cells to the inflamed organs. Real time PCR analysis indicated that PD-L1 blockade substantially up-regulated CXCL9 and -10 gene expression in the SMG of NOD/ShiLtJ mice (Fig. 4A), which may at least in part contribute to the increase in T cells in the SMG by promoting their tissue-infiltration. Hence, the disease-accelerating effect of PD-L1 blockade is accompanied by increased Th1/Tc1 chemoattractants. Furthermore, consistent with an increase in B cells in the SMG shown by flow cytometric analysis, mRNA levels of B cell marker CD19 and B cell chemoattractant CXCL13 in the SMG were significantly increased by PD-L1 blockade (Fig. 4B). Assessment of several cytokines that can promote B cell function and antibody production showed that the mRNA levels of B cell activating factor, IL-21 and IL-13 were not affected by PD-L1 blockade (Fig. 4B, and data not shown). Hence, the disease-accelerating effect of anti-PD-L1 is associated with increased amounts of B cells and CXCL13 in the SMG. In summary, PD-L1 blockade increases the expression of Th1/Tc1 signature molecules and T- and B-cell chemoattractants in the SMG.

### Local administration of exogenous IFN-γ into the SMG causes impaired salivary secretion

**Figure 5:**
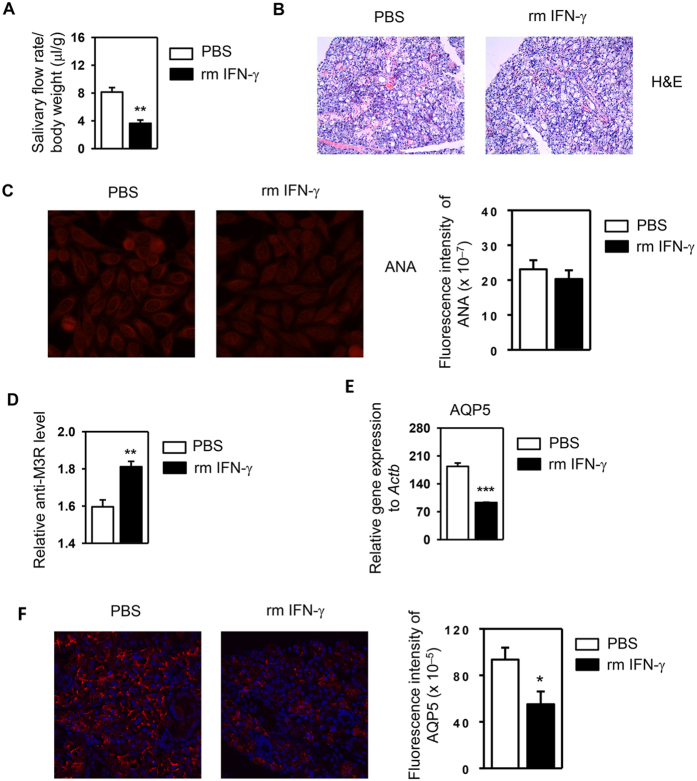
Local administration of exogenous IFN-γ into the SMG causes impaired salivary secretion. Recombinant mouse IFN-γ was directly injected into the SMG of NOD/ShiLtJ mice every 3 days for a total of 3 times. (**A**) Stimulated saliva flow rate normalized to body weight. (**B**) H&E staining of SMG sections. Original magnification ×200. (**C**) Detection of serum ANA and the quantification of the fluorescence intensity of ANA staining (right panel). Original magnification: ×400. (**D**) Serum anti- M3R autoantibody level was determined by ELISA. (**E**) Real-time PCR analysis of relative AQP5 mRNA levels in the SMG. (**F**) Immunofluorescence staining of AQP5 protein in SMG sections and the quantification of the fluorescence intensity of AQP5 staining (right panel). Data are representative or the average of the analyses of 4 mice for each group. *P < 0.05, **P < 0.01; ***P < 0.001.

Genetic deficiency of IFN-γ in NOD/ShiLtJ mice prevents the development of SS-like pathologies including SMG tissue inflammation and apoptosis, ANA production, and hyposalivation, indicating a crucial requirement for IFN-γ in the development of SS. Since PD-L1 blockade led to enhanced Th1 and Tc1 responses and increased IFN-γ production, we reasoned that the excess IFN-γ may account for the disease-acceleration induced by PD-L1 blockade. To test this hypothesis, we injected 3 μg of recombinant murine (rm) IFN-γ or control PBS directly into the SMG of 6 week-old female NOD/ShiLtJ mice every 3 days for a total of 3 times, and analyzed the SS disease profile. Treatment with rm IFN-γ significantly reduced the stimulated saliva flow rate in NOD/ShiLtJ mice (Fig. 5A). IFN-γ treatment did not promote leukocyte infiltration or caspase-3 activation in SMG tissues (Fig. 5B, and data not shown). Interestingly, IFN-γ treatment did not affect serum ANA levels (Fig. 5C), but increased the levels of anti-M3R autoantibodies, which have the ability to impair normal salivary secretion (Fig. 5D)^50^,^51^. To further define additional mechanisms of IFN-γ effect on salivary secretion, we examined the expression of aquaporin 5 (AQP5), a water channel protein critical for normal salivary secretion^52^,^53^,^54^. IFN-γ treatment reduced AQP5 mRNA amounts in the SMG (Fig. 5E) and decreased AQP5 protein levels as determined by immunofluorescence staining (Fig. 5F). Hence, intra-SMG administration of IFN-γ leads to impaired saliva secretion in NOD/ShiLtJ mice, which is accompanied by reduced AQP5 expression and augmented anti-M3R production. These results indicate that the increased IFN-γ expression caused by PD-L1 blockade may be responsible for the exacerbated secretory dysfunction but not for other pathological changes induced by this treatment.

### Neutralization of IFN-γ improves salivary secretion and AQP5 expression in anti-PD-L1-treated NOD/ShiLtJ mice

**Figure 6:**
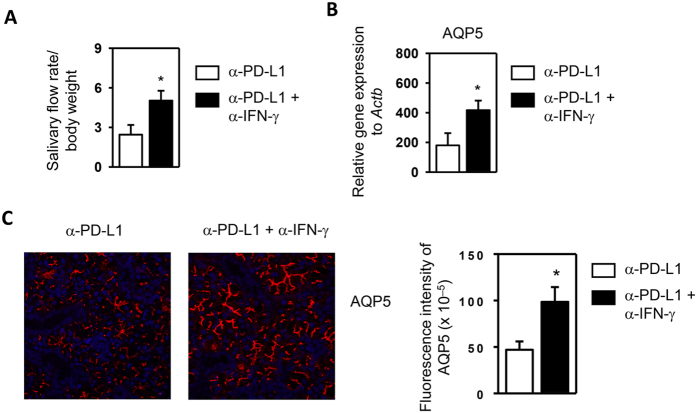
Neutralization of IFN-γ improves salivary secretion in anti-PD-L1-treated NOD/ShiLtJ mice. 6 week-old female NOD/ShiLtJ mice were *i.p*.-injected with 200 μg of rat IgG1 or anti-IFN-γ together with 200 μg of anti-PD-L1 every other day for a total of 4 times. (**A**) Stimulated saliva flow rate normalized to body weight. (**B**) Real-time PCR analysis of relative AQP5 mRNA levels in the SMG. (**C**) Immunofluorescence staining of AQP5 protein in SMG sections, and the quantification of the fluorescence intensity of AQP5 staining (right panel). Original magnification: ×400. Data are representative or the average of the analyses of 8 mice for each group.

Having shown that exogenous IFN-γ administration impairs salivary secretion, we next determined whether IFN-γ blockade can improve salivary secretion in anti-PD-L1-treated NOD/ShiLtJ mice. We *i.p*.-injected the anti-PD-L1 antibody to NOD/ShiLtJ mice in conjunction with a neutralizing anti-IFN-γ antibody or the isotype control IgG. Our results demonstrated that anti-IFN-γ markedly improved the salivary flow rate (Fig. 6A). Real-time PCR analysis and immunofluorescence staining demonstrated that anti-IFN-γ treatment markedly increased AQP5 mRNA and protein levels in the SMG (Fig. 6B and C). IFN-γ neutralization did not reduce the inflammation of SMG or the levels of serum autoantibodies (Data not shown). Hence, increased amount of IFN-γ upon PD-L1 blockade down-regulates AQP5 production and contributes to the salivary gland secretory dysfunction.

In summary, we propose a model in which the interaction between PD-L1 and PD-1 impedes the recruitment of Th1 and Tc1 to the salivary glands in part by down-regulating CXCL9. Reduced IFN-γ production resulting from impaired local Th1/Tc1 responses in turn increases AQP5 expression and diminishes anti-M3R autoantibody production, thereby hindering the development of salivary gland hypofunction. In addition, PD-L1-PD-1 interaction also impedes B cell recruitment to the salivary glands in part by down-regulating CXCL13, and thereby curtails B cell activation and autoantibody production. Hence, endogenous PD-L1, which is up-regulated during early phase of SS development, impedes the development and delays the onset of SS in a negative feedback fashion (Supplementary Figure S4).

## Discussion

In the present study, we demonstrate a protective role of endogenous PD-L1 in the development and onset of SS-like disease in NOD mice and elucidate the cellular mechanisms underlying its immune-regulatory function in this disease.

Salivary gland-infiltrating lymphocytes in SS patients express higher levels of PD-1 than those from control subjects and PD-L1 is expressed in salivary gland epithelial cells in most of the patients^35^. Consistent with these findings, we showed that PD-L1 and PD-1 expression in the salivary glands of NOD/ShiLtJ mice increases considerably, accompanying the development of SS. Hence, up-regulation of this inhibitory pathway, possibly as a result of production of the inflammatory mediators at this stage, acts as a negative feedback mechanism to impede the development and onset of this disease. The cellular sources of PD-1 and PD-L1 in this study may include activated T and B cells, Tregs, salivary gland epithelial cells and APCs. The most well-characterized interaction between PD-L1 and PD-1 in autoimmune and inflammatory conditions is the one between PD-L1 expressed on tissue cells or APCs and PD-1 expressed on T cells^25^,^35^,^38^,^43^. Additionally, PD-L1 expressed by activated T cells^24^ as well as by Tregs^29^,^55^,^56^ can also engage PD-1 to suppress the activation and function of T cells. The precise cellular sources of PD-L1 critical for the inhibition of SS development in the NOD/ShiLtJ mice require further determination by specifically abolishing PD-L1 expression in these cell populations.

SS have a strong female propensity, with 90% of the patients being women^50^,^57^. It has been well-documented that female NOD mice mostly develop spontaneous autoimmune sialadenitis, whereas male NOD mice mostly develop autoimmune dacryoadenitis (dry eyes)^58^,^59^. Our recent analysis of the disease profiles in both female and male NOD/ShiLtJ mice also confirmed these findings. In this study, we only used female mice to study the effect of PD-L1 blockade on SS-like sialadenitis, which has the same gender propensity in both human patients and NOD mice. However, it will be important to assess whether PD-L1 blockade can similarly accelerate the development and onset of SS-like lacrimal gland inflammation and dysfunction using male NOD mice. Furthermore, our future investigations will also assess whether enhancing PD-L1-PD-1 activity with exogenous PD-L1-Fc can prevent the development and onset of SS, and reverse or attenuate the established SS in both male and female NOD mice.

In this study, we administered anti-PD-L1 antibody repeatedly into NOD/ShiLtJ mice between 6 to 8 weeks of age. Previous reports have shown that most of NOD/ShiLtJ mice become diabetic between 12 and 16 weeks of age, and the development and onset of the type-1 diabetes is accelerated upon PD-L1 blockade^37^,^60^. We therefore monitored the urine glucose levels in mice during the course of anti-PD-L1 treatment and found that they remained normal in all the mice throughout the experiments. Therefore, the acceleration of SS development induced by PD-L1 blockade in the present study is not a secondary consequence of clinical diabetes.

Consistent with the findings in a number of other autoimmune disorders^24^,^25^,^46^,^47^, here we showed that PD-L1 blockade and preferentially enhances Th1/Tc1 responses and IFN-γ production, without affecting Th2 and Th17 responses, in the SS disease setting. It has been reported that IFN-γ-gene-deficiency prevents the development of pathologies of SS and the secretory dysfunction in NOD/ShiLtJ mice^17^. Here we show that indeed, administration of IFN-γ replicates, whereas neutralization of IFN-γ attenuates the detrimental effect of PD-L1 blockade on salivary gland secretory function. The effect of IFN-γ correlates with the alteration in the level of AQP5, a key water channel protein for normal salivary secretion^52^,^53^,^54^. AQP5 deficiency leads to significantly reduced salivary volume due to defective water export from the salivary epithelial cells^52^,^53^, and local gene therapy that overexpresses AQP1 to compensates for defective AQP5 expression restores salivary secretion in a mouse model of SS^54^. It is interesting that IFN-γ administration leads to increased production of anti-M3R antibody, which has a reported effect of impairing the salivary gland cell secretory function in response to parasympathetic neurotransmitters^50^,^51^. It is not clear to us how it affects this specific autoantibody without affecting the ANA production and future investigations will be needed to elucidate the underlying mechanisms. We also find that excess IFN-γ in the SMG does not replicate, and neutralization of IFN-γ does not mitigate the accelerating effect of PD-L1 blockade on leukocyte infiltration, ANA production and tissue apoptosis. Hence, the excessive IFN-γ production is likely a consequence of enhanced Th1/Tc1 response in the SMG resulting from PD-L1 blockade, which in turn promotes the salivary gland secretory dysfunction possibly by inhibiting AQP5 expression and enhancing anti-M3R production. It is not essentially required for the exacerbation of other pathological events induced by PD-L1 blockade.

Another intriguing finding in this study is that anti-PD-L1 treatment increases the expression level of Foxp3, the key transcription factor of Treg cells. PD-L1 promotes the development and sustains the suppressive function of Treg cells by regulating Foxp3^61^, and PD-L1 expressed by Tregs constitutes as one of the pivotal mechanisms by which Tregs exert their suppressive effect^29^,^55^,^56^. However, a recent report demonstrates that PD-L1 can also negatively regulate the expansion of Treg cells in the context of chronic viral inflammation^62^, supporting the notion that the effect of PD-L1 on Tregs is disease context-dependent. Hence, the specific effect of PD-L1 on Treg expansion and function in the SS setting needs to be characterized in greater depth in future. Furthermore, the specific characteristics of Tregs and their functional importance in the SS disease setting also await investigation by using mouse models.

In addition to autoreactive T cell responses, B cell infiltration of exocrine glands and their activation and autoantibody production are also characteristic events in SS that are crucial to the full onset of salivary gland secretory dysfunction^9^,^63^,^64^. We found that the number of B cells in the salivary gland of NOD/ShiLtJ mice and the production of ANA and anti-M3R antibodies are significantly enhanced by PD-L1 blockade. Although several reports have shown that PD-L1 can directly affect B cell activation and proliferation^65^,^66^, we did not observe a direct effect of PD-L1 on B cell expansion and antibody production in *in vitro* cultures (Data not shown). We therefore hypothesize that PD-L1 indirectly suppresses B cell function and autoantibody production by inhibiting T cell responses, in particular, Th1/Tc1 response and IFN-γ production. Indeed, in both SS patients and mouse SS models, T cell infiltration of target organs precedes B cell infiltration^4^,^64^. Moreover, deletion or inhibition of T cell cytokines IFN-γ and IL-17 prevents the development of all major pathologies of SS including B cell infiltration and ANA production^17^,^67^. These findings highlight the crucial role of T cell response for the induction of subsequent autoreactive B cell response. Hence, it is plausible that by inhibiting T cell activation and cytokine production, PD-L1 can suppress the subsequent B cell activation and antibody production. One specific event induced by anti-PD-L1 treatment is the up-regulation of B cell chemoattractant CXCL13, which is elevated in a number of autoimmune diseases including SS^68^,^69^. Importantly, studies in both human patients and mouse models strongly suggest CXCL13 as a biomarker and an essential pathogenic player in SS^69^. Here, we showed that PD-L1 blockade leads to elevated CXCL13 expression in the salivary glands, which may promote B cell recruitment and the consequent production of autoantibodies. Future studies will investigate the main cellular sources of CXCL13 and the mechanisms of enhanced CXCL13 production as a result of PD-L1 blockade in the SS disease context.

## Conclusions

The present study demonstrated that PD-L1 expression is up-regulated in the salivary glands of female NOD mice during the developmental phase of SS, which in turn hinders the development and onset of this disease in a negative feedback fashion. Thus, enhancing the activities PD-L1-dependent pathways may be an effective therapeutic strategy to combat this disease for which no effective treatment is currently available.

## Methods

### Mice

Female non-obese diabetic (NOD/ShiLtJ) mice were purchased from the Jackson Laboratory and were kept under specific pathogen-free conditions. All experimental protocols were approved by the Institutional Animal Care and Use Committee of the Forsyth Institute. All methods were carried out in accordance with the National Institutes of Health guidelines for the care and use of laboratory animals.

### Antibodies, peptides and cytokines

Purified monoclonal anti-mouse PD-L1 (10F.9G2), anti-mouse IFN-γ (XMG1.2), and isotype control rat IgG2b and rat IgG1 were purchased from BioXCell. Recombinant mouse (rm) IFN-γ was purchased from Peprotech. The muscarinic acetycholine receptor (M3R) peptide was synthesized by Biomatik Corporation. For Flow cytometry, anti-CD4, anti-CD8 and anti-CD44 antibodies were purchased from BioLegend. For immunohistological chemistry, biotin conjugated anti-CD4 antibody was obtained from eBioscience, anti-T-bet and biotin conjugated anti-B220 antibodies were from BioLegend, and biotin conjugated anti-rat IgG2bwere purchased from Vector Laboratories. For immunofluorescence staining, anti-aquaporin 5 (AQP5) and Alexa Fluor647-conjugated anti-rabbit IgG antibodies were purchased from Abcam.

### *In vivo* administration of anti-PD-L1 antibody, IFN-γ and anti-IFN-γ antibody

Female NOD/ShiLtJ mice were *i.p.*-injected with 200 μg of IgG or anti-PD-L1 every other day for a total of 4 times, starting from 6 weeks of age. For IFN-γ administration, 6 week-old female NOD/ShiLtJ mice were anesthetized with ketamine hydrochloride/xylazine hydrochloride and 3 μg rm IFN-γ was injected directly into the submandibular gland (SMG) every 3 days for a total of 3 times. For anti-IFN-γ antibody administration, 6 week-old female NOD/ShiLtJ mice were *i.p*.-injected with 200 μg anti-PD-L1, together with 200 μg rat IgG1 or anti-IFN-γ, every other day for a total of 4 times. All the analyses were performed 2 days after the last injection.

### Histological analysis

SMG tissue samples were fixed in 4% paraformaldehyde, embedded in paraffin and sectioned to 5 μm thickness. Sections were then stained with hematoxylin and eosin (H&E) and examined for leukocytic infiltration. The number of leukocytic foci in each of the three non-consecutive sections of each SMG sample was counted, and the highest number among the three was used for further calculation and statistical analysis.

### Immunofluorescence staining

Rehydrated SMG sections were subjected to antigen unmasking process and then incubated with rabbit anti-mouse AQP5 antibody at 4 °C overnight, followed by Alexa Fluor647-conjugated anti-rabbit IgG for 1 h at room temperature. The stained samples were examined and imaged (magnification: ×400) under a Leica laser scanning confocal microscope (Zeiss, Oberkochen, Germany). Quantification of the fluorescence intensity was performed using ImageJ 1.50i software. Briefly, images were converted into grayscale (8-bit) and the grey color was segmented using thresholding. Integrated density measurement was performed to determine the fluorescence intensity of the staining.

### Immunohistochemical staining

SMG sections were subjected to de-paraffinization and stained with anti-mouse CD4, anti-mouse T-bet, or anti-mouse B220 antibody at 4 °C overnight using VECTASTAIN Elite ABC Kit (Vector Laboratories) following the manufacturer’s instructions. Active caspase-3 was detected by SignalStain® Apoptosis (Cleaved Caspase-3) IHC Detection Kit (Cell Signaling Technology), according to the manufacturer’s manual. The stained samples were examined and imaged (magnification ×400) under a light microscope. Quantification of the positively-stained cells in the samples was performed using ImageJ 1.50i software. Briefly, images were saved as RGB Tiff files and only the brown-stained cells were segmented using appropriate color thresholding. The total number of the thresholded cells, which were positively-stained cells, in each sample was measured and calculated.

### Detection of serum antinuclear antibodies (ANA)

ANA in mouse sera were detected using Alexa Fluor568-conjugated anti-mouse IgG (ThermoFisher Scientific) and HEp-2 human epithelial cell substrate slides (INOVA Diagnostics) following the manufacturer’s instructions. The stained samples were examined with inverted wide-field fluorescence microscope (Zeiss) at a magnification of 400X. Images presented were processed using Zeiss software (ZEN blue edition). Quantification of the fluorescence intensity was performed using ImageJ 1.50i software as described. Integrated density measurement was performed to determine the fluorescence intensity of the staining.

### Measurement of salivary flow rate

Non-anesthetized mice were weighed and given an *i.p.*- injection of 100 μl PBS-based secretagogue solution containing isoproterenol (0.02 mg/ml) and pilocarpine (0.05 mg/ml). One min after secretagogue injection, saliva was collected continuously for 5 min from the oral cavity of mice with a micropipette. The volume of saliva was measured and normalized to the body weight.

### RNA isolation and Real-time RT-PCR

SMG lobes were cut into small fragments and grinded into single cells using frosted glass slides. The total SMG cells were resuspended in RNA lysis buffer and subjected to RNA isolation using RNeasy Micro kit (Qiagen) followed by reverse transcription with M-MLV reverse transcriptase (Promega). The resulting cDNA was subjected to SYBR Green-based real-time PCR amplification (Qiagen) for 40 cycles with annealing and extension temperature at 60 °C, on a LightCycler 480 Real-Time PCR System (Roche). Primer sequences are: mouse PD-L1 forward, 5′-GGTGCGGACTACAAGCGAAT-3′, reverse, 5′-TTCATGCTCAGAAGTGGCTGG-3′; PD-1 forward, 5′-AAATCGAGGAGAGCCCTGGA-3′, reverse, 5′-CATGCCTTGAAACCGGCCTT-3′; T-bet forward, 5′-CCAACAACCCCTTTGCCAAAG-3′, reverse, 5′-TCCCCCAAGCAGTTGACAGT-3′; IFN-γ forward, 5′-GGATGCATTCATGAGTATTGC-3′, reverse, 5′-CTTTTCCGCTTCCTGAGG-3′; TNF-α forward, 5′-CCTTTCACTCACTGGCCCAA-3′, reverse, 5′-AGTGCCTCTTCTGCCAGTTC-3′. Other sequences will be provided upon request. The relative mRNA level of each gene relative to that of β-actin was analyzed using the LightCycler^@^480 software.

### ELISA

The M3R peptide solution, at 2 μg/ml in the ELISA coating buffer (Biolegend), was adsorbed onto a Nunc™ MaxiSorp™ flat-bottom 96 well plate overnight at 4 °C. Non-specific binding sites on the plate were then blocked by incubating with ELISA assay diluent buffer (Biolegend) for 1 h at room temperature. Mouse sera (1:6 diluted in the blocking buffer) were added to the plate and incubated overnight at 4 °C. The plate was washed and incubated with biotinlated goat anti-mouse IgG antibody (Vector Labratories) at 1:300 dilution for 1 h at room temperature. After washing, the bound antibodies were detected by incubation with Avidin-HRP solution for 30 min, followed by incubation with TMB substrate solution. Finally, the absorbance was read on a microplate reader (BioTek) at 450 nm.

### Statistical analysis

All statistical significance was determined by Student’s t-test (two-tailed, two sample equal variance). P values smaller than 0.05 were considered as statistically significant.

## Additional Information

**How to cite this article**: Zhou, J. *et al*. Endogenous programmed death ligand-1 restrains the development of Sjögren’s syndrome in non-obese diabetic mice. *Sci. Rep.* **6**, 39105; doi: 10.1038/srep39105 (2016).

**Publisher's note:** Springer Nature remains neutral with regard to jurisdictional claims in published maps and institutional affiliations.

## Supplementary Material

Supplementary Materials
