# Supplementary material for: Endogenous programmed death ligand-1 restrains the development and onset of Sjӧgren’s syndrome in non-obese diabetic mice
Source: Sci Rep. 2016 Dec 14;6:39105. doi: 10.1038/srep39105 (PMC5155421; doi:10.1038/srep39105)
Supplement: Supplementary Materials [file srep39105-s1.pdf]

Supplementary Materials

Endogenous programmed death ligand-1 restrains the development and onset of Sjögren's syndrome in non-obese diabetic mice

Jing Zhou[a,b], Jun-O Jin[a,1], Toshihisa Kawai[a,b] and Qing Yu[a,b,*]

[a] Department of Immunology and Infectious Diseases, the Forsyth Institute,
245 First Street, Cambridge, MA 02142, USA
[b] Department of Oral Medicine, Infection and Immunity, Harvard School of Dental Medicine,
188 Longwood Avenue, Boston, MA 02115, USA
[1] Current affiliation: Shanghai Public Health Clinical Center, Shanghai Medical College, Fudan University, Shanghai, 201508, China

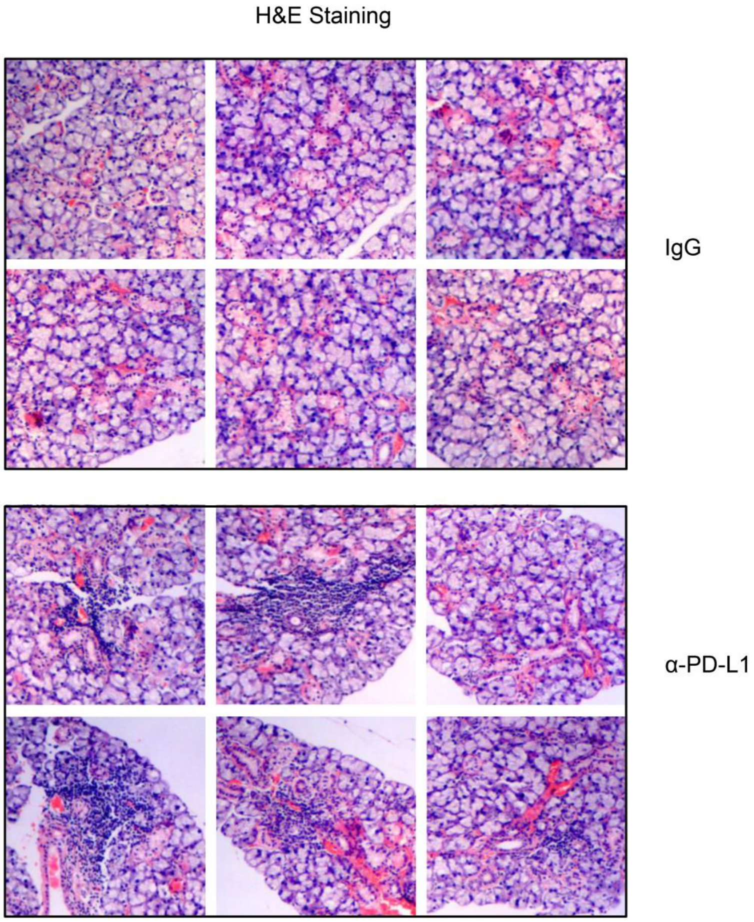

Supplementary Figure S1. Blockade of PD-L1 accelerates the development of salivary gland inflammation. NOD/ShiLtJ mice received injections of rat anti-mouse PD-L1 antibody and the isotype control rat IgG2b. Each group of tile image shows multiple areas of a representative H&E stained SMG section from the IgG-treated group (upper panel) or anti-PD-L1-treated group (lower panel). Original magnification × 200. Data are representative of analyses of 18 - 20 mice for each group.

Table S1. Blockade of PD-L1 accelerates leukocyte infiltration of the SMG

Mouse \ Treatment	IgG	anti-PD-L1
**1**	0	0
**2**	0	12
**3**	0	0
**4**	0	6
**5**	0	6
**6**	0	1
**7**	1	0
**8**	0	0
**9**	0	4
**10**	0	0
**11**	0	0
**12**	0	0
**13**	0	1
**14**	0	7
**15**	0	0
**16**	0	0
**17**	0	4
**18**	0	5
**19**		1
**20**		2
**% Mice with focus (foci)**	5.5	55
**Mean number of foci**	0.055	2.45 ($P<0.05$)

Supplementary Table S1. Blockade of PD-L1 accelerates the appearance of leukocyte foci in the SMG. The number of leukocyte foci, defined as leukocyte aggregates containing at least 50 cells in a 4 mm^2 area of SMG sections, in NOD/ShiLtJ mice treated with anti-PD-L1 or the isotype control IgG.

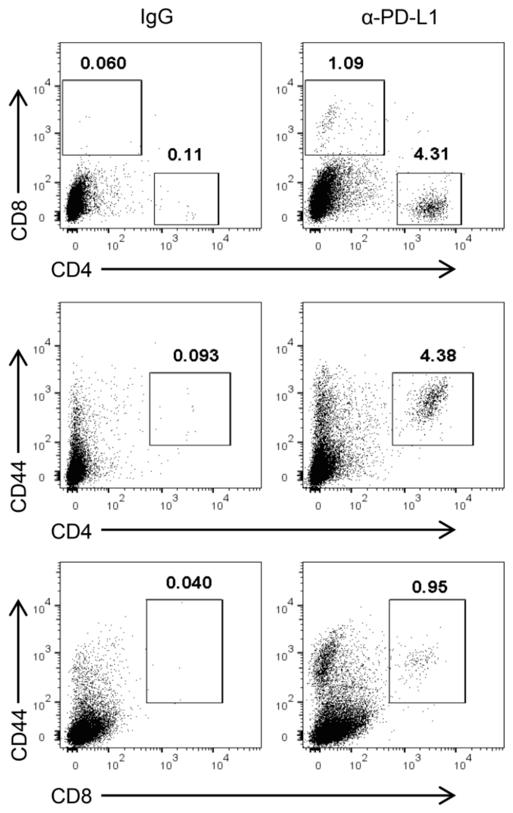

Supplementary Figure S2. PD-L1 blockade increases the number of CD4 and CD8 T cells in the SMG. Representative flow cytometry of T lymphocyte populations in SMG-infiltrating mononuclear cells. Data are representative of 6-7 mice for each group.

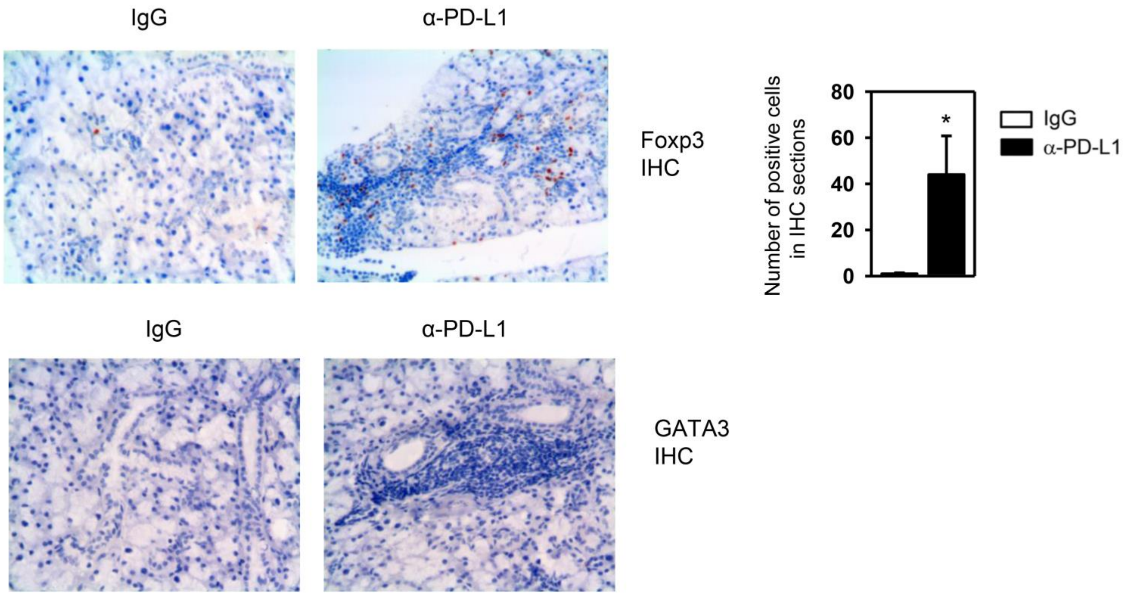

Supplementary Figure S3. PD-L1 blockade increases the number of Tregs in the SMG. Immunohistochemical staining of Foxp3 (upper panels) and GATA3 (lower panels) in the SMG sections of anti-PD-L1- and IgG-treated NOD/ShiLtJ mice. Original magnification: × 400. The number of $Foxp3^{+}$ cells in the immunohisto-chemistry sections were quantified and shown (upper right panel). Data are representative of or the average of the analyses of 6-7 mice for each group. Error bars represent the SEM. *P < 0.05

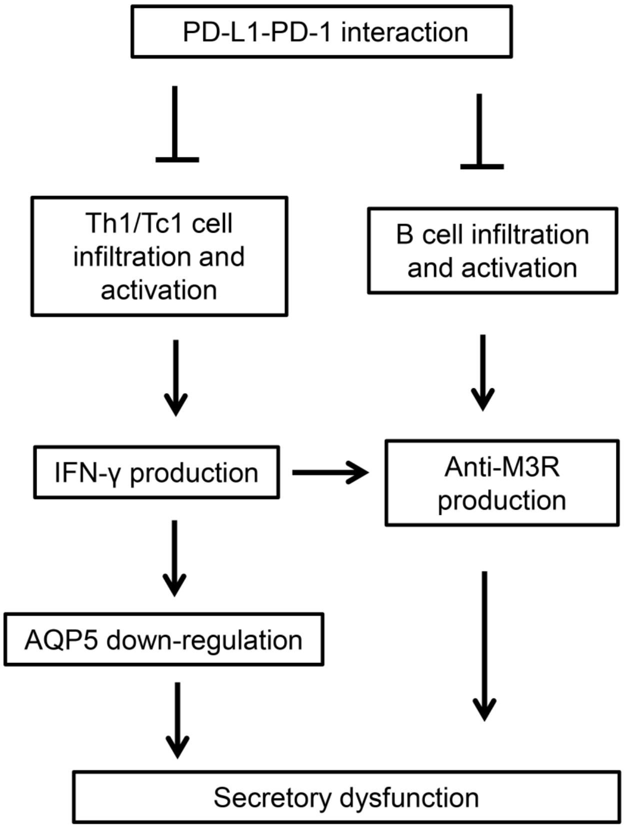

Supplementary Figure S4. Proposed mechanisms of the regulatory effect of PD-L1 on SS development. The interaction between PD-L1 and PD-1 impedes Th1 and Tc1 recruitment to the salivary glands in part by down-regulating CXCL9. Diminshed IFN-γ production resulting from impaired Th1 and Tc1 responses in turn increases AQP5 expression and reduces anti-M3R autoantibody production to hinder the development of salivary gland hypofunction. In addition, PD-L1-PD-1 interaction also impedes B cell recruitment in part by down-regulating CXCL13, thereby curtailing B cell activation and autoantibody production. Hence, endogenous PD-L1, which is up-regulated during early phase of SS development, impedes the development and delays the onset of this disease in a negative feedback manner.
